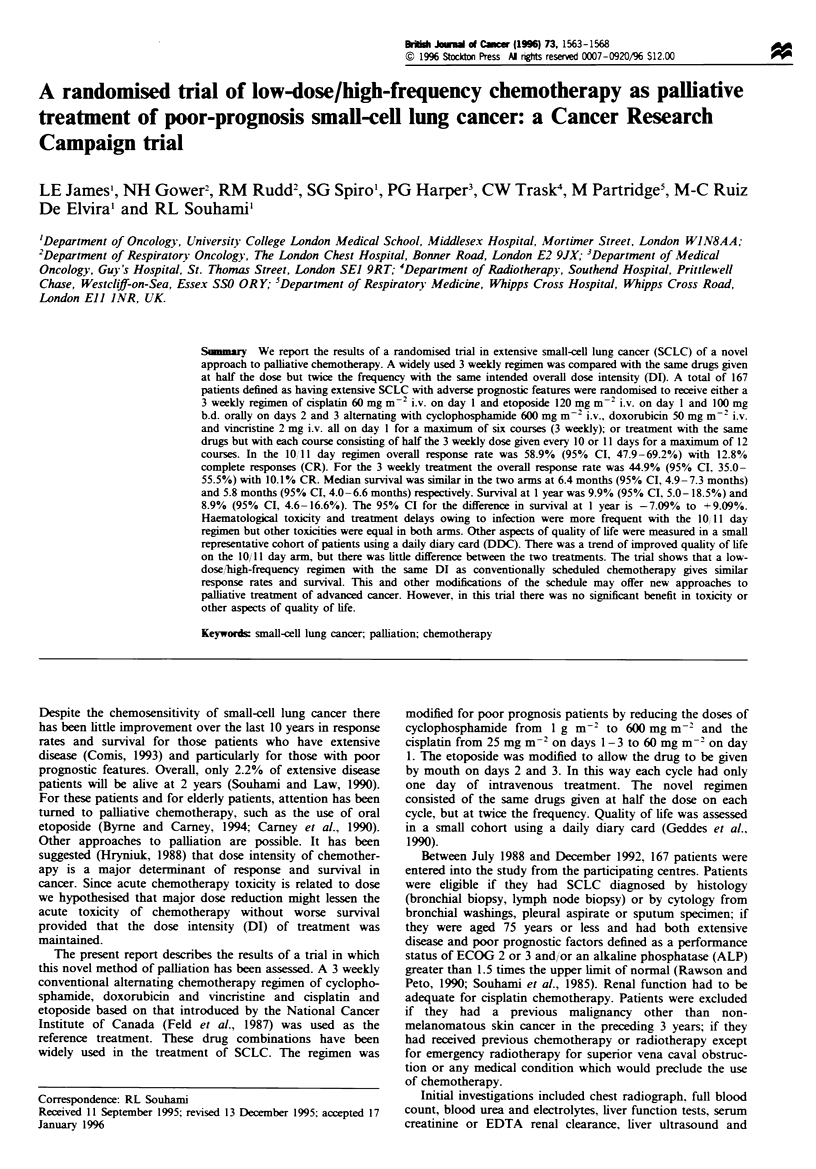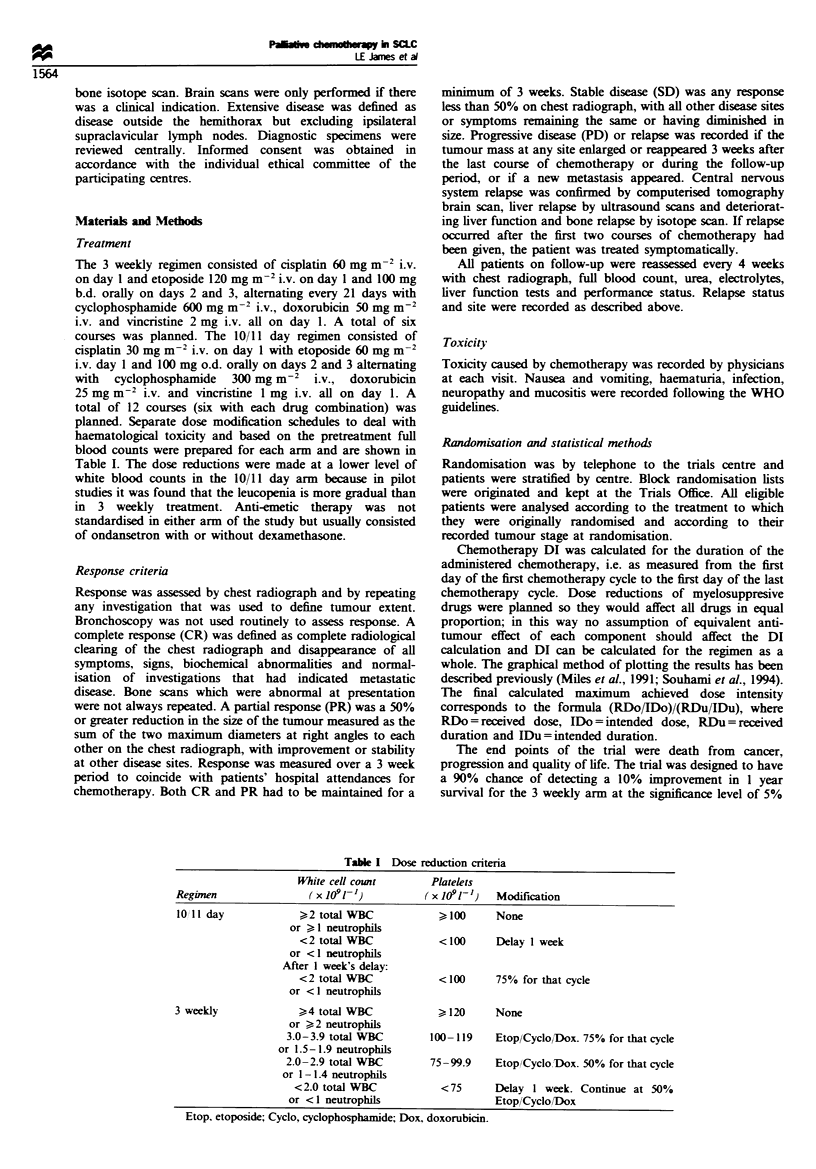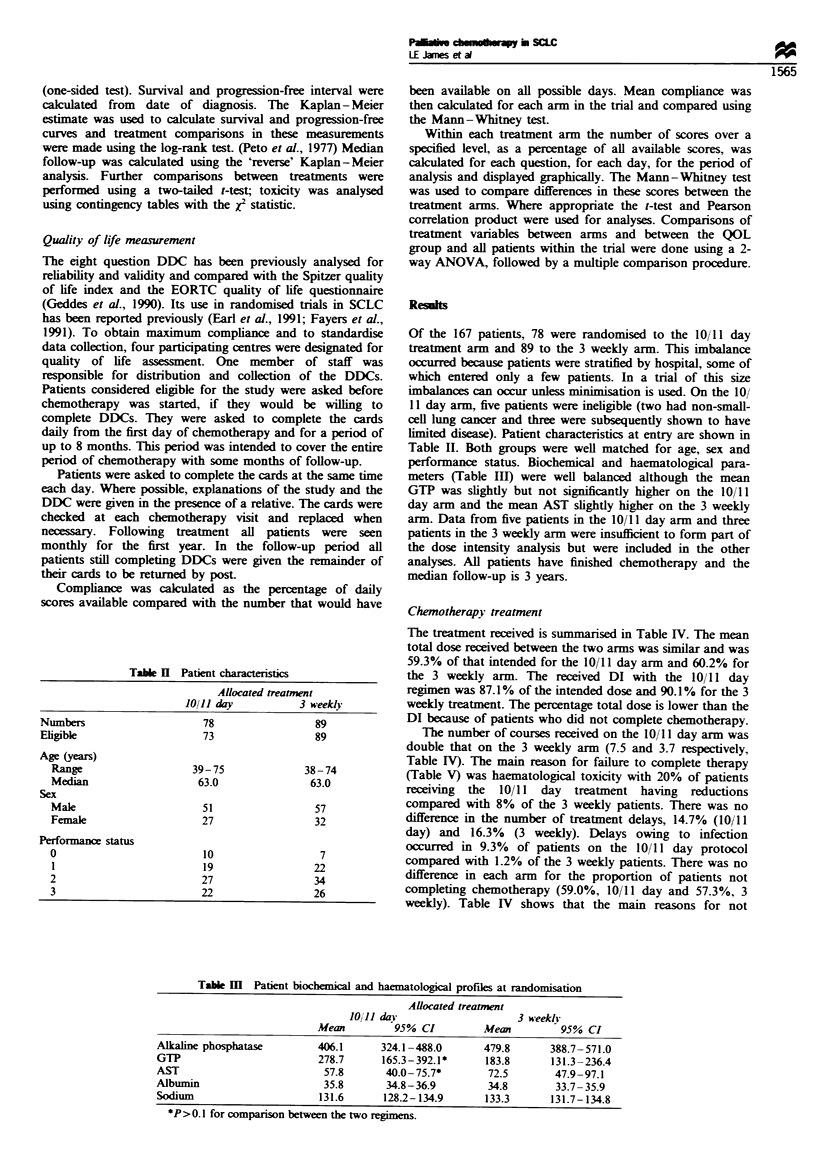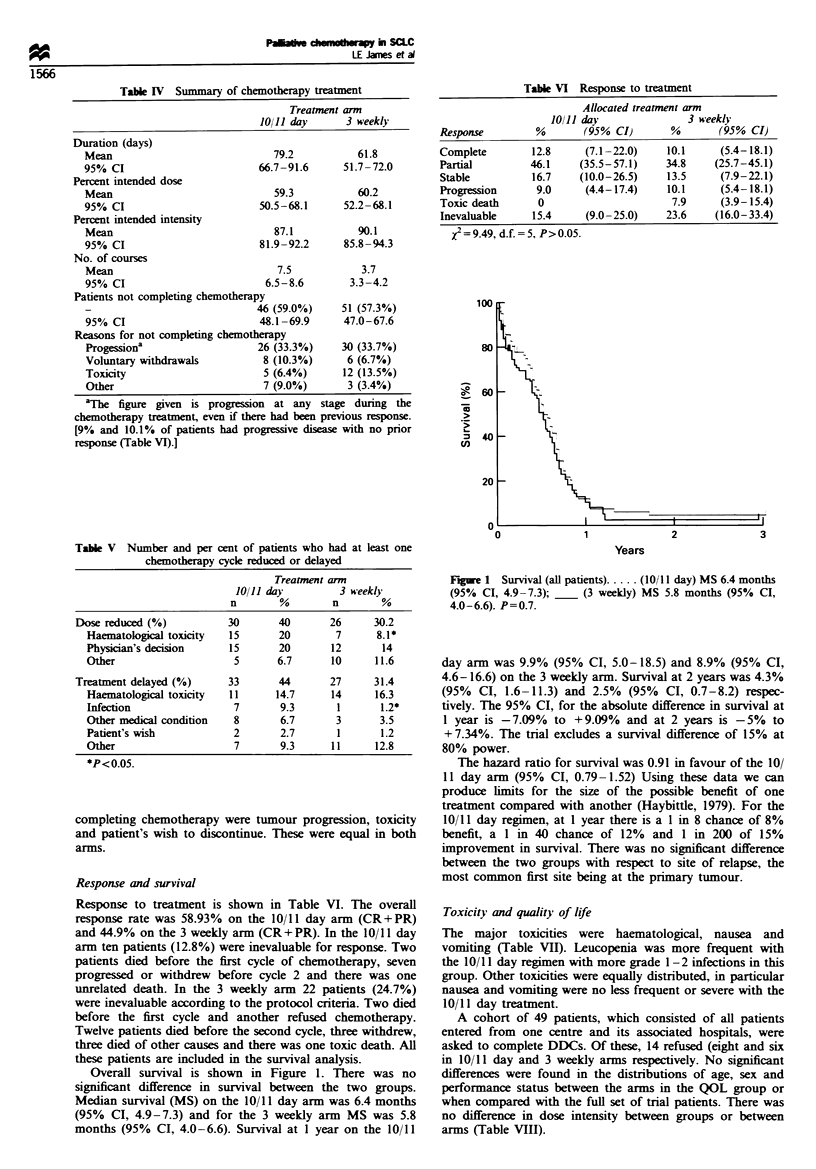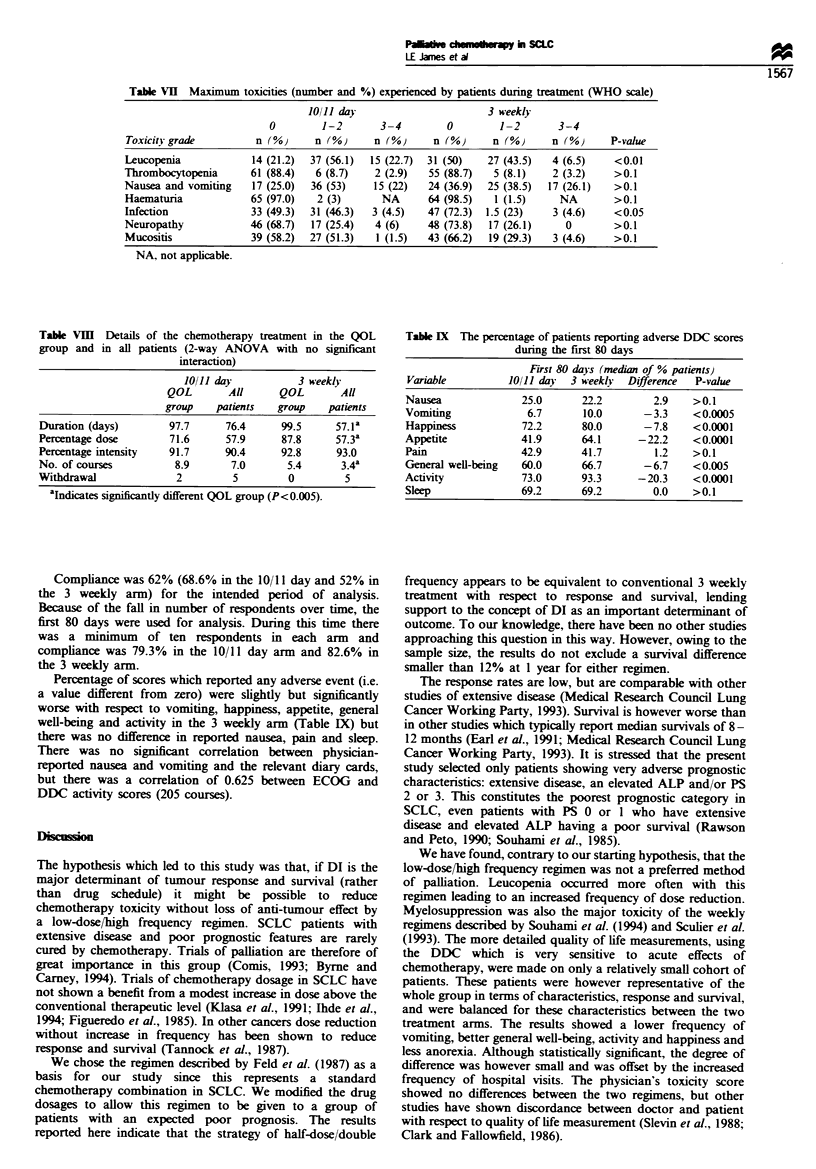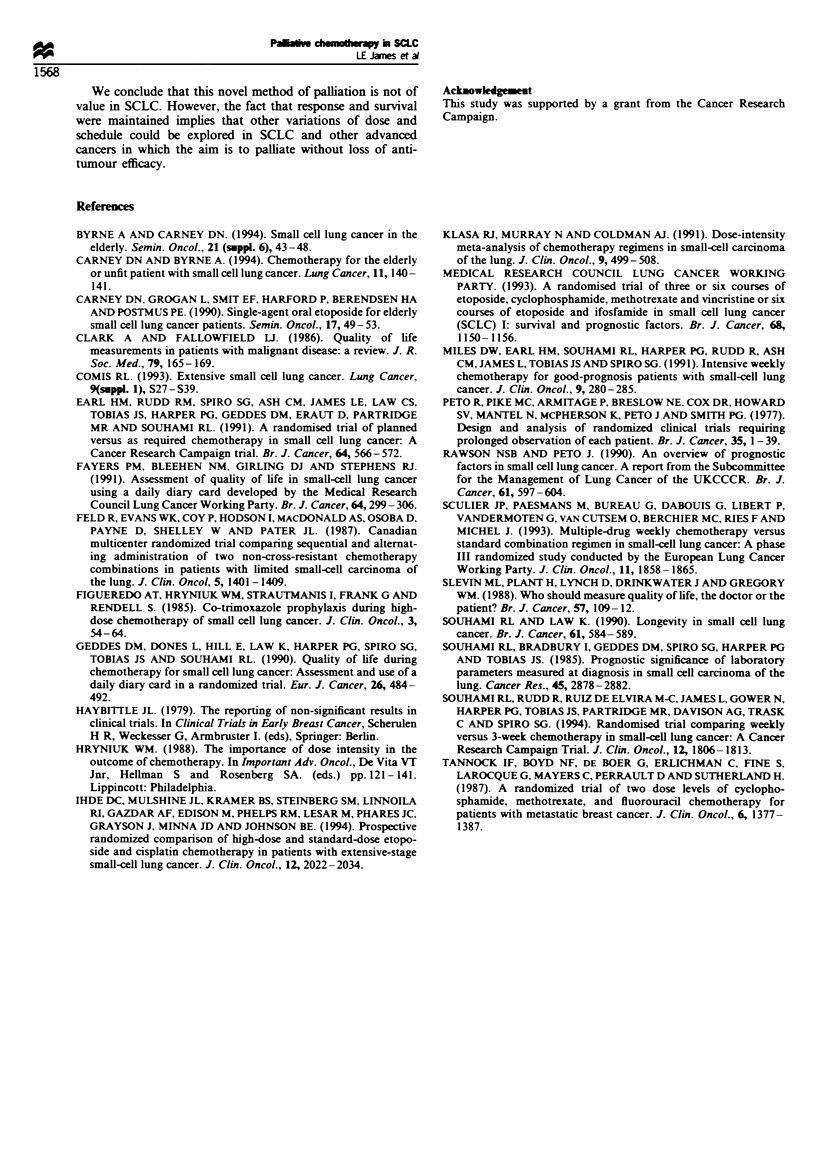# A randomised trial of low-dose/high-frequency chemotherapy as palliative treatment of poor-prognosis small-cell lung cancer: a Cancer research Campaign trial.

**DOI:** 10.1038/bjc.1996.295

**Published:** 1996-06

**Authors:** L. E. James, N. H. Gower, R. M. Rudd, S. G. Spiro, P. G. Harper, C. W. Trask, M. Partridge, M. C. Ruiz de Elvira, R. L. Souhami

**Affiliations:** Department of Oncology, University College London Medical School, Middlesex Hospital, UK.

## Abstract

We report the results of a randomised trial in extensive small-cell lung cancer (SCLC) of a novel approach to palliative chemotherapy. A widely used 3 weekly regimen was compared with the same drugs given at half the dose but twice the frequency with the same intended overall dose intensity (DI). A total of 167 patients defined as having extensive SCLC with adverse prognostic features were randomised to receive either a 3 weekly regimen of cisplatin 60 mg m-2 i.v. on day 1 and etoposide 120 mg m-2 i.v. on day 1 and 100 mg b.d. orally on days 2 and 3 alternating with cyclophosphamide 600 mg m-2 i.v., doxorubicin 50 mg m-2 i.v. and vincristine 2 mg i.v. all on day 1 for a maximum of six courses (3 weekly); or treatment with the same drugs but with each course consisting of half the 3 weekly dose given every 10 or 11 days for a maximum of 12 courses. In the 10/11 day regimen overall response rate was 58.9% (95% CI, 47.9-69.2%) with 12.8% complete responses (CR). For the 3 weekly treatment the overall response rate was 44.9% (95% CI, 35.0-55.5%) with 10.1% CR. Median survival was similar in the two arms at 6.4 months (95% CI, 4.9-7.3 months) and 5.8 months (95% CI, 4.0-6.6 months) respectively. Survival at 1 year was 9.9% (95% CI, 5.0-18.5%) and 8.9% (95% CI, 4.6-16.6%). The 95% CI for the difference in survival at 1 year is -7.09% to +9.09%. Haematological toxicity and treatment delays owing to infection were more frequent with the 10/11 day regimen but other toxicities were equal in both arms. Other aspects of quality of life were measured in a small representative cohort of patients using a daily diary card (DDC). There was a trend of improved quality of life on the 10/11 day arm, but there was little difference between the two treatments. The trial shows that a low-dose/high-frequency regimen with the same DI as conventionally scheduled chemotherapy gives similar response rates and survival. This and other modifications of the schedule may offer new approaches to palliative treatment of advanced cancer. However, in this trial there was no significant benefit in toxicity or other aspects of quality of life.


British Journal of Cancer (1996) 73, 1563-1568

© 1996 Stockton Press All rights reserved 0007-0920/96 $12.00

### A randomised trial of low-dose/high-frequency chemotherapy as palliative treatment of poor-prognosis small-cell lung cancer: a Cancer Research Campaign trial

LE James[1], NH Gower[2], RM Rudd[2], SG Spiro[1], PG Harper[3], CW Trask[4], M Partridge[5], M-C Ruiz De Elvira[1] and RL Souhami[1]

[1]*Department of Oncology, University College London Medical School, Middlesex Hospital, Mortimer Street, London W1N8AA;* [2]*Department of Respiratory Oncology, The London Chest Hospital, Bonner Road, London E2 9JX;* [3]*Department of Medical Oncology, Guy's Hospital, St. Thomas Street, London SE1 9RT;* [4]*Department of Radiotherapy, Southend Hospital, Prittlewell Chase, Westcliff-on-Sea, Essex SS0 0RY;* [5]*Department of Respiratory Medicine, Whipps Cross Hospital, Whipps Cross Road, London E11 1NR, UK.*

**Summary** We report the results of a randomised trial in extensive small-cell lung cancer (SCLC) of a novel approach to palliative chemotherapy. A widely used 3 weekly regimen was compared with the same drugs given at half the dose but twice the frequency with the same intended overall dose intensity (DI). A total of 167 patients defined as having extensive SCLC with adverse prognostic features were randomised to receive either a 3 weekly regimen of cisplatin 60 mg $m^{-2}$ i.v. on day 1 and etoposide 120 mg $m^{-2}$ i.v. on day 1 and 100 mg b.d. orally on days 2 and 3 alternating with cyclophosphamide 600 mg $m^{-2}$ i.v., doxorubicin 50 mg $m^{-2}$ i.v. and vincristine 2 mg i.v. all on day 1 for a maximum of six courses (3 weekly); or treatment with the same drugs but with each course consisting of half the 3 weekly dose given every 10 or 11 days for a maximum of 12 courses. In the 10/11 day regimen overall response rate was 58.9% (95% CI, 47.9-69.2%) with 12.8% complete responses (CR). For the 3 weekly treatment the overall response rate was 44.9% (95% CI, 35.0-55.5%) with 10.1% CR. Median survival was similar in the two arms at 6.4 months (95% CI, 4.9-7.3 months) and 5.8 months (95% CI, 4.0-6.6 months) respectively. Survival at 1 year was 9.9% (95% CI, 5.0-18.5%) and 8.9% (95% CI, 4.6-16.6%). The 95% CI for the difference in survival at 1 year is −7.09% to +9.09%. Haematological toxicity and treatment delays owing to infection were more frequent with the 10/11 day regimen but other toxicities were equal in both arms. Other aspects of quality of life were measured in a small representative cohort of patients using a daily diary card (DDC). There was a trend of improved quality of life on the 10/11 day arm, but there was little difference between the two treatments. The trial shows that a low-dose/high-frequency regimen with the same DI as conventionally scheduled chemotherapy gives similar response rates and survival. This and other modifications of the schedule may offer new approaches to palliative treatment of advanced cancer. However, in this trial there was no significant benefit in toxicity or other aspects of quality of life.

**Keywords:** small-cell lung cancer; palliation; chemotherapy

Despite the chemosensitivity of small-cell lung cancer there has been little improvement over the last 10 years in response rates and survival for those patients who have extensive disease (Comis, 1993) and particularly for those with poor prognostic features. Overall, only 2.2% of extensive disease patients will be alive at 2 years (Souhami and Law, 1990). For these patients and for elderly patients, attention has been turned to palliative chemotherapy, such as the use of oral etoposide (Byrne and Carney, 1994; Carney *et al.*, 1990). Other approaches to palliation are possible. It has been suggested (Hryniuk, 1988) that dose intensity of chemotherapy is a major determinant of response and survival in cancer. Since acute chemotherapy toxicity is related to dose we hypothesised that major dose reduction might lessen the acute toxicity of chemotherapy without worse survival provided that the dose intensity (DI) of treatment was maintained.

The present report describes the results of a trial in which this novel method of palliation has been assessed. A 3 weekly conventional alternating chemotherapy regimen of cyclophosphamide, doxorubicin and vincristine and cisplatin and etoposide based on that introduced by the National Cancer Institute of Canada (Feld *et al.*, 1987) was used as the reference treatment. These drug combinations have been widely used in the treatment of SCLC. The regimen was modified for poor prognosis patients by reducing the doses of cyclophosphamide from 1 g $m^{-2}$ to 600 mg $m^{-2}$ and the cisplatin from 25 mg $m^{-2}$ on days 1-3 to 60 mg $m^{-2}$ on day 1. The etoposide was modified to allow the drug to be given by mouth on days 2 and 3. In this way each cycle had only one day of intravenous treatment. The novel regimen consisted of the same drugs given at half the dose on each cycle, but at twice the frequency. Quality of life was assessed in a small cohort using a daily diary card (Geddes *et al.*, 1990).

Between July 1988 and December 1992, 167 patients were entered into the study from the participating centres. Patients were eligible if they had SCLC diagnosed by histology (bronchial biopsy, lymph node biopsy) or by cytology from bronchial washings, pleural aspirate or sputum specimen; if they were aged 75 years or less and had both extensive disease and poor prognostic factors defined as a performance status of ECOG 2 or 3 and/or an alkaline phosphatase (ALP) greater than 1.5 times the upper limit of normal (Rawson and Peto, 1990; Souhami *et al.*, 1985). Renal function had to be adequate for cisplatin chemotherapy. Patients were excluded if they had a previous malignancy other than non-melanomatous skin cancer in the preceding 3 years; if they had received previous chemotherapy or radiotherapy except for emergency radiotherapy for superior vena caval obstruction or any medical condition which would preclude the use of chemotherapy.

Initial investigations included chest radiograph, full blood count, blood urea and electrolytes, liver function tests, serum creatinine or EDTA renal clearance, liver ultrasound and

Correspondence: RL Souhami
Received 11 September 1995; revised 13 December 1995; accepted 17 January 1996

bone isotope scan. Brain scans were only performed if there was a clinical indication. Extensive disease was defined as disease outside the hemithorax but excluding ipsilateral supraclavicular lymph nodes. Diagnostic specimens were reviewed centrally. Informed consent was obtained in accordance with the individual ethical committee of the participating centres.

### Materials and Methods

### *Treatment*

The 3 weekly regimen consisted of cisplatin 60 mg $m^{-2}$ i.v. on day 1 and etoposide 120 mg $m^{-2}$ i.v. on day 1 and 100 mg b.d. orally on days 2 and 3, alternating every 21 days with cyclophosphamide 600 mg $m^{-2}$ i.v., doxorubicin 50 mg $m^{-2}$ i.v. and vincristine 2 mg i.v. all on day 1. A total of six courses was planned. The 10/11 day regimen consisted of cisplatin 30 mg $m^{-2}$ i.v. on day 1 with etoposide 60 mg $m^{-2}$ i.v. day 1 and 100 mg o.d. orally on days 2 and 3 alternating with cyclophosphamide 300 mg $m^{-2}$ i.v., doxorubicin 25 mg $m^{-2}$ i.v. and vincristine 1 mg i.v. all on day 1. A total of 12 courses (six with each drug combination) was planned. Separate dose modification schedules to deal with haematological toxicity and based on the pretreatment full blood counts were prepared for each arm and are shown in Table I. The dose reductions were made at a lower level of white blood counts in the 10/11 day arm because in pilot studies it was found that the leucopenia is more gradual than in 3 weekly treatment. Anti-emetic therapy was not standardised in either arm of the study but usually consisted of ondansetron with or without dexamethasone.

### *Response criteria*

Response was assessed by chest radiograph and by repeating any investigation that was used to define tumour extent. Bronchoscopy was not used routinely to assess response. A complete response (CR) was defined as complete radiological clearing of the chest radiograph and disappearance of all symptoms, signs, biochemical abnormalities and normalisation of investigations that had indicated metastatic disease. Bone scans which were abnormal at presentation were not always repeated. A partial response (PR) was a 50% or greater reduction in the size of the tumour measured as the sum of the two maximum diameters at right angles to each other on the chest radiograph, with improvement or stability at other disease sites. Response was measured over a 3 week period to coincide with patients' hospital attendances for chemotherapy. Both CR and PR had to be maintained for a minimum of 3 weeks. Stable disease (SD) was any response less than 50% on chest radiograph, with all other disease sites or symptoms remaining the same or having diminished in size. Progressive disease (PD) or relapse was recorded if the tumour mass at any site enlarged or reappeared 3 weeks after the last course of chemotherapy or during the follow-up period, or if a new metastasis appeared. Central nervous system relapse was confirmed by computerised tomography brain scan, liver relapse by ultrasound scans and deteriorating liver function and bone relapse by isotope scan. If relapse occurred after the first two courses of chemotherapy had been given, the patient was treated symptomatically.

All patients on follow-up were reassessed every 4 weeks with chest radiograph, full blood count, urea, electrolytes, liver function tests and performance status. Relapse status and site were recorded as described above.

### *Toxicity*

Toxicity caused by chemotherapy was recorded by physicians at each visit. Nausea and vomiting, haematuria, infection, neuropathy and mucositis were recorded following the WHO guidelines.

### *Randomisation and statistical methods*

Randomisation was by telephone to the trials centre and patients were stratified by centre. Block randomisation lists were originated and kept at the Trials Office. All eligible patients were analysed according to the treatment to which they were originally randomised and according to their recorded tumour stage at randomisation.

Chemotherapy DI was calculated for the duration of the administered chemotherapy, i.e. as measured from the first day of the first chemotherapy cycle to the first day of the last chemotherapy cycle. Dose reductions of myelosuppresive drugs were planned so they would affect all drugs in equal proportion; in this way no assumption of equivalent antitumour effect of each component should affect the DI calculation and DI can be calculated for the regimen as a whole. The graphical method of plotting the results has been described previously (Miles *et al.*, 1991; Souhami *et al.*, 1994). The final calculated maximum achieved dose intensity corresponds to the formula (RDo/IDo)/(RDu/IDu), where RDo = received dose, IDo = intended dose, RDu = received duration and IDu = intended duration.

The end points of the trial were death from cancer, progression and quality of life. The trial was designed to have a 90% chance of detecting a 10% improvement in 1 year survival for the 3 weekly arm at the significance level of 5%

**Table I** Dose reduction criteria

| Regimen | *White cell count* ( $\times 10^9 l^{-1}$ ) | *Platelets* ( $\times 10^9 l^{-1}$ ) | Modification |
|---|---|---|---|
| 10/11 day | ≥2 total WBC or ≥1 neutrophils | ≥100 | None |
| | <2 total WBC or <1 neutrophils | <100 | Delay 1 week |
| | After 1 week's delay: <2 total WBC or <1 neutrophils | <100 | 75% for that cycle |
| 3 weekly | ≥4 total WBC or ≥2 neutrophils | ≥120 | None |
| | 3.0–3.9 total WBC or 1.5–1.9 neutrophils | 100–119 | Etop/Cyclo/Dox. 75% for that cycle |
| | 2.0–2.9 total WBC or 1–1.4 neutrophils | 75–99.9 | Etop/Cyclo/Dox. 50% for that cycle |
| | <2.0 total WBC or <1 neutrophils | <75 | Delay 1 week. Continue at 50% Etop/Cyclo/Dox |

Etop, etoposide; Cyclo, cyclophosphamide; Dox, doxorubicin.

(one-sided test). Survival and progression-free interval were calculated from date of diagnosis. The Kaplan-Meier estimate was used to calculate survival and progression-free curves and treatment comparisons in these measurements were made using the log-rank test. (Peto et al., 1977) Median follow-up was calculated using the 'reverse' Kaplan-Meier analysis. Further comparisons between treatments were performed using a two-tailed $t$-test; toxicity was analysed using contingency tables with the $\chi^2$ statistic.

*Quality of life measurement*

The eight question DDC has been previously analysed for reliability and validity and compared with the Spitzer quality of life index and the EORTC quality of life questionnaire (Geddes et al., 1990). Its use in randomised trials in SCLC has been reported previously (Earl et al., 1991; Fayers et al., 1991). To obtain maximum compliance and to standardise data collection, four participating centres were designated for quality of life assessment. One member of staff was responsible for distribution and collection of the DDCs. Patients considered eligible for the study were asked before chemotherapy was started, if they would be willing to complete DDCs. They were asked to complete the cards daily from the first day of chemotherapy and for a period of up to 8 months. This period was intended to cover the entire period of chemotherapy with some months of follow-up.

Patients were asked to complete the cards at the same time each day. Where possible, explanations of the study and the DDC were given in the presence of a relative. The cards were checked at each chemotherapy visit and replaced when necessary. Following treatment all patients were seen monthly for the first year. In the follow-up period all patients still completing DDCs were given the remainder of their cards to be returned by post.

Compliance was calculated as the percentage of daily scores available compared with the number that would have been available on all possible days. Mean compliance was then calculated for each arm in the trial and compared using the Mann-Whitney test.

Within each treatment arm the number of scores over a specified level, as a percentage of all available scores, was calculated for each question, for each day, for the period of analysis and displayed graphically. The Mann-Whitney test was used to compare differences in these scores between the treatment arms. Where appropriate the $t$-test and Pearson correlation product were used for analyses. Comparisons of treatment variables between arms and between the QOL group and all patients within the trial were done using a 2-way ANOVA, followed by a multiple comparison procedure.

**Table II** Patient characteristics

| | *Allocated treatment* 10/11 day | 3 weekly |
|---|---|---|
| Numbers | 78 | 89 |
| Eligible | 73 | 89 |
| Age (years) | | |
| Range | 39-75 | 38-74 |
| Median | 63.0 | 63.0 |
| Sex | | |
| Male | 51 | 57 |
| Female | 27 | 32 |
| Performance status | | |
| 0 | 10 | 7 |
| 1 | 19 | 22 |
| 2 | 27 | 34 |
| 3 | 22 | 26 |

### Results

Of the 167 patients, 78 were randomised to the 10/11 day treatment arm and 89 to the 3 weekly arm. This imbalance occurred because patients were stratified by hospital, some of which entered only a few patients. In a trial of this size imbalances can occur unless minimisation is used. On the 10/11 day arm, five patients were ineligible (two had non-small-cell lung cancer and three were subsequently shown to have limited disease). Patient characteristics at entry are shown in Table II. Both groups were well matched for age, sex and performance status. Biochemical and haematological parameters (Table III) were well balanced although the mean GTP was slightly but not significantly higher on the 10/11 day arm and the mean AST slightly higher on the 3 weekly arm. Data from five patients in the 10/11 day arm and three patients in the 3 weekly arm were insufficient to form part of the dose intensity analysis but were included in the other analyses. All patients have finished chemotherapy and the median follow-up is 3 years.

*Chemotherapy treatment*

The treatment received is summarised in Table IV. The mean total dose received between the two arms was similar and was 59.3% of that intended for the 10/11 day arm and 60.2% for the 3 weekly arm. The received DI with the 10/11 day regimen was 87.1% of the intended dose and 90.1% for the 3 weekly treatment. The percentage total dose is lower than the DI because of patients who did not complete chemotherapy.

The number of courses received on the 10/11 day arm was double that on the 3 weekly arm (7.5 and 3.7 respectively, Table IV). The main reason for failure to complete therapy (Table V) was haematological toxicity with 20% of patients receiving the 10/11 day treatment having reductions compared with 8% of the 3 weekly patients. There was no difference in the number of treatment delays, 14.7% (10/11 day) and 16.3% (3 weekly). Delays owing to infection occurred in 9.3% of patients on the 10/11 day protocol compared with 1.2% of the 3 weekly patients. There was no difference in each arm for the proportion of patients not completing chemotherapy (59.0%, 10/11 day and 57.3%, 3 weekly). Table IV shows that the main reasons for not

**Table III** Patient biochemical and haematological profiles at randomisation

| | *Allocated treatment* 10/11 day | | 3 weekly | |
|---|---|---|---|---|
| | Mean | 95% CI | Mean | 95% CI |
| Alkaline phosphatase | 406.1 | 324.1-488.0 | 479.8 | 388.7-571.0 |
| GTP | 278.7 | 165.3-392.1* | 183.8 | 131.3-236.4 |
| AST | 57.8 | 40.0-75.7* | 72.5 | 47.9-97.1 |
| Albumin | 35.8 | 34.8-36.9 | 34.8 | 33.7-35.9 |
| Sodium | 131.6 | 128.2-134.9 | 133.3 | 131.7-134.8 |

*$P > 0.1$ for comparison between the two regimens.

Table IV Summary of chemotherapy treatment

| | Treatment arm 10/11 day | 3 weekly |
|---|---|---|
| Duration (days) | | |
| Mean | 79.2 | 61.8 |
| 95% CI | 66.7-91.6 | 51.7-72.0 |
| Percent intended dose | | |
| Mean | 59.3 | 60.2 |
| 95% CI | 50.5-68.1 | 52.2-68.1 |
| Percent intended intensity | | |
| Mean | 87.1 | 90.1 |
| 95% CI | 81.9-92.2 | 85.8-94.3 |
| No. of courses | | |
| Mean | 7.5 | 3.7 |
| 95% CI | 6.5-8.6 | 3.3-4.2 |
| Patients not completing chemotherapy | | |
| - | 46 (59.0%) | 51 (57.3%) |
| 95% CI | 48.1-69.9 | 47.0-67.6 |
| Reasons for not completing chemotherapy | | |
| Progession[a] | 26 (33.3%) | 30 (33.7%) |
| Voluntary withdrawals | 8 (10.3%) | 6 (6.7%) |
| Toxicity | 5 (6.4%) | 12 (13.5%) |
| Other | 7 (9.0%) | 3 (3.4%) |

[a]The figure given is progression at any stage during the chemotherapy treatment, even if there had been previous response. [9% and 10.1% of patients had progressive disease with no prior response (Table VI).]

Table V Number and per cent of patients who had at least one chemotherapy cycle reduced or delayed

| | Treatment arm 10/11 day n | % | 3 weekly n | % |
|---|---|---|---|---|
| Dose reduced (%) | 30 | 40 | 26 | 30.2 |
| Haematological toxicity | 15 | 20 | 7 | 8.1* |
| Physician's decision | 15 | 20 | 12 | 14 |
| Other | 5 | 6.7 | 10 | 11.6 |
| Treatment delayed (%) | 33 | 44 | 27 | 31.4 |
| Haematological toxicity | 11 | 14.7 | 14 | 16.3 |
| Infection | 7 | 9.3 | 1 | 1.2* |
| Other medical condition | 8 | 6.7 | 3 | 3.5 |
| Patient's wish | 2 | 2.7 | 1 | 1.2 |
| Other | 7 | 9.3 | 11 | 12.8 |

*$P<0.05$.

completing chemotherapy were tumour progression, toxicity and patient's wish to discontinue. These were equal in both arms.

*Response and survival*

Response to treatment is shown in Table VI. The overall response rate was 58.93% on the 10/11 day arm (CR+PR) and 44.9% on the 3 weekly arm (CR+PR). In the 10/11 day arm ten patients (12.8%) were inevaluable for response. Two patients died before the first cycle of chemotherapy, seven progressed or withdrew before cycle 2 and there was one unrelated death. In the 3 weekly arm 22 patients (24.7%) were inevaluable according to the protocol criteria. Two died before the first cycle and another refused chemotherapy. Twelve patients died before the second cycle, three withdrew, three died of other causes and there was one toxic death. All these patients are included in the survival analysis.

Overall survival is shown in Figure 1. There was no significant difference in survival between the two groups. Median survival (MS) on the 10/11 day arm was 6.4 months (95% CI, 4.9-7.3) and for the 3 weekly arm MS was 5.8 months (95% CI, 4.0-6.6). Survival at 1 year on the 10/11

Table VI Response to treatment

| Response | Allocated treatment arm 10/11 day % | (95% CI) | 3 weekly % | (95% CI) |
|---|---|---|---|---|
| Complete | 12.8 | (7.1-22.0) | 10.1 | (5.4-18.1) |
| Partial | 46.1 | (35.5-57.1) | 34.8 | (25.7-45.1) |
| Stable | 16.7 | (10.0-26.5) | 13.5 | (7.9-22.1) |
| Progression | 9.0 | (4.4-17.4) | 10.1 | (5.4-18.1) |
| Toxic death | 0 | | 7.9 | (3.9-15.4) |
| Inevaluable | 15.4 | (9.0-25.0) | 23.6 | (16.0-33.4) |

$\chi^2=9.49$, d.f.$=5$, $P>0.05$.

Figure 1 Survival (all patients)..... (10/11 day) MS 6.4 months (95% CI, 4.9-7.3); ____ (3 weekly) MS 5.8 months (95% CI, 4.0-6.6). $P=0.7$.

day arm was 9.9% (95% CI, 5.0-18.5) and 8.9% (95% CI, 4.6-16.6) on the 3 weekly arm. Survival at 2 years was 4.3% (95% CI, 1.6-11.3) and 2.5% (95% CI, 0.7-8.2) respectively. The 95% CI, for the absolute difference in survival at 1 year is -7.09% to +9.09% and at 2 years is -5% to +7.34%. The trial excludes a survival difference of 15% at 80% power.

The hazard ratio for survival was 0.91 in favour of the 10/11 day arm (95% CI, 0.79-1.52) Using these data we can produce limits for the size of the possible benefit of one treatment compared with another (Haybittle, 1979). For the 10/11 day regimen, at 1 year there is a 1 in 8 chance of 8% benefit, a 1 in 40 chance of 12% and 1 in 200 of 15% improvement in survival. There was no significant difference between the two groups with respect to site of relapse, the most common first site being at the primary tumour.

*Toxicity and quality of life*

The major toxicities were haematological, nausea and vomiting (Table VII). Leucopenia was more frequent with the 10/11 day regimen with more grade 1-2 infections in this group. Other toxicities were equally distributed, in particular nausea and vomiting were no less frequent or severe with the 10/11 day treatment.

A cohort of 49 patients, which consisted of all patients entered from one centre and its associated hospitals, were asked to complete DDCs. Of these, 14 refused (eight and six in 10/11 day and 3 weekly arms respectively. No significant differences were found in the distributions of age, sex and performance status between the arms in the QOL group or when compared with the full set of trial patients. There was no difference in dose intensity between groups or between arms (Table VIII).

Table VII Maximum toxicities (number and %) experienced by patients during treatment (WHO scale)

| | 10/11 day | | | 3 weekly | | | |
|---|---|---|---|---|---|---|---|
| | 0 | 1-2 | 3-4 | 0 | 1-2 | 3-4 | |
| *Toxicity grade* | n (%) | n (%) | n (%) | n (%) | n (%) | n (%) | P-*value* |
| Leucopenia | 14 (21.2) | 37 (56.1) | 15 (22.7) | 31 (50) | 27 (43.5) | 4 (6.5) | <0.01 |
| Thrombocytopenia | 61 (88.4) | 6 (8.7) | 2 (2.9) | 55 (88.7) | 5 (8.1) | 2 (3.2) | >0.1 |
| Nausea and vomiting | 17 (25.0) | 36 (53) | 15 (22) | 24 (36.9) | 25 (38.5) | 17 (26.1) | >0.1 |
| Haematuria | 65 (97.0) | 2 (3) | NA | 64 (98.5) | 1 (1.5) | NA | >0.1 |
| Infection | 33 (49.3) | 31 (46.3) | 3 (4.5) | 47 (72.3) | 1.5 (23) | 3 (4.6) | <0.05 |
| Neuropathy | 46 (68.7) | 17 (25.4) | 4 (6) | 48 (73.8) | 17 (26.1) | 0 | >0.1 |
| Mucositis | 39 (58.2) | 27 (51.3) | 1 (1.5) | 43 (66.2) | 19 (29.3) | 3 (4.6) | >0.1 |

NA, not applicable.

Table VIII Details of the chemotherapy treatment in the QOL group and in all patients (2-way ANOVA with no significant interaction)

| | 10/11 day | | 3 weekly | |
|---|---|---|---|---|
| | *QOL group* | *All patients* | *QOL group* | *All patients* |
| Duration (days) | 97.7 | 76.4 | 99.5 | 57.1[a] |
| Percentage dose | 71.6 | 57.9 | 87.8 | 57.3[a] |
| Percentage intensity | 91.7 | 90.4 | 92.8 | 93.0 |
| No. of courses | 8.9 | 7.0 | 5.4 | 3.4[a] |
| Withdrawal | 2 | 5 | 0 | 5 |

[a]Indicates significantly different QOL group ($P<0.005$).

Table IX The percentage of patients reporting adverse DDC scores during the first 80 days

| | First 80 days (median of % patients) | | | |
|---|---|---|---|---|
| *Variable* | *10/11 day* | *3 weekly* | *Difference* | P-*value* |
| Nausea | 25.0 | 22.2 | 2.9 | >0.1 |
| Vomiting | 6.7 | 10.0 | −3.3 | <0.0005 |
| Happiness | 72.2 | 80.0 | −7.8 | <0.0001 |
| Appetite | 41.9 | 64.1 | −22.2 | <0.0001 |
| Pain | 42.9 | 41.7 | 1.2 | >0.1 |
| General well-being | 60.0 | 66.7 | −6.7 | <0.005 |
| Activity | 73.0 | 93.3 | −20.3 | <0.0001 |
| Sleep | 69.2 | 69.2 | 0.0 | >0.1 |

Compliance was 62% (68.6% in the 10/11 day and 52% in the 3 weekly arm) for the intended period of analysis. Because of the fall in number of respondents over time, the first 80 days were used for analysis. During this time there was a minimum of ten respondents in each arm and compliance was 79.3% in the 10/11 day arm and 82.6% in the 3 weekly arm.

Percentage of scores which reported any adverse event (i.e. a value different from zero) were slightly but significantly worse with respect to vomiting, happiness, appetite, general well-being and activity in the 3 weekly arm (Table IX) but there was no difference in reported nausea, pain and sleep. There was no significant correlation between physician-reported nausea and vomiting and the relevant diary cards, but there was a correlation of 0.625 between ECOG and DDC activity scores (205 courses).

### Discussion

The hypothesis which led to this study was that, if DI is the major determinant of tumour response and survival (rather than drug schedule) it might be possible to reduce chemotherapy toxicity without loss of anti-tumour effect by a low-dose/high frequency regimen. SCLC patients with extensive disease and poor prognostic features are rarely cured by chemotherapy. Trials of palliation are therefore of great importance in this group (Comis, 1993; Byrne and Carney, 1994). Trials of chemotherapy dosage in SCLC have not shown a benefit from a modest increase in dose above the conventional therapeutic level (Klasa *et al.*, 1991; Ihde *et al.*, 1994; Figueredo *et al.*, 1985). In other cancers dose reduction without increase in frequency has been shown to reduce response and survival (Tannock *et al.*, 1987).

We chose the regimen described by Feld *et al.* (1987) as a basis for our study since this represents a standard chemotherapy combination in SCLC. We modified the drug dosages to allow this regimen to be given to a group of patients with an expected poor prognosis. The results reported here indicate that the strategy of half-dose/double frequency appears to be equivalent to conventional 3 weekly treatment with respect to response and survival, lending support to the concept of DI as an important determinant of outcome. To our knowledge, there have been no other studies approaching this question in this way. However, owing to the sample size, the results do not exclude a survival difference smaller than 12% at 1 year for either regimen.

The response rates are low, but are comparable with other studies of extensive disease (Medical Research Council Lung Cancer Working Party, 1993). Survival is however worse than in other studies which typically report median survivals of 8–12 months (Earl *et al.*, 1991; Medical Research Council Lung Cancer Working Party, 1993). It is stressed that the present study selected only patients showing very adverse prognostic characteristics: extensive disease, an elevated ALP and/or PS 2 or 3. This constitutes the poorest prognostic category in SCLC, even patients with PS 0 or 1 who have extensive disease and elevated ALP having a poor survival (Rawson and Peto, 1990; Souhami *et al.*, 1985).

We have found, contrary to our starting hypothesis, that the low-dose/high frequency regimen was not a preferred method of palliation. Leucopenia occurred more often with this regimen leading to an increased frequency of dose reduction. Myelosuppression was also the major toxicity of the weekly regimens described by Souhami *et al.* (1994) and Sculier *et al.* (1993). The more detailed quality of life measurements, using the DDC which is very sensitive to acute effects of chemotherapy, were made on only a relatively small cohort of patients. These patients were however representative of the whole group in terms of characteristics, response and survival, and were balanced for these characteristics between the two treatment arms. The results showed a lower frequency of vomiting, better general well-being, activity and happiness and less anorexia. Although statistically significant, the degree of difference was however small and was offset by the increased frequency of hospital visits. The physician's toxicity score showed no differences between the two regimens, but other studies have shown discordance between doctor and patient with respect to quality of life measurement (Slevin *et al.*, 1988; Clark and Fallowfield, 1986).

We conclude that this novel method of palliation is not of value in SCLC. However, the fact that response and survival were maintained implies that other variations of dose and schedule could be explored in SCLC and other advanced cancers in which the aim is to palliate without loss of anti-tumour efficacy.

**Acknowledgement**
This study was supported by a grant from the Cancer Research Campaign.

### References

BYRNE A AND CARNEY DN. (1994). Small cell lung cancer in the elderly. *Semin. Oncol.*, **21 (suppl. 6),** 43–48.

CARNEY DN AND BYRNE A. (1994). Chemotherapy for the elderly or unfit patient with small cell lung cancer. *Lung Cancer*, **11,** 140–141.

CARNEY DN, GROGAN L, SMIT EF, HARFORD P, BERENDSEN HA AND POSTMUS PE. (1990). Single-agent oral etoposide for elderly small cell lung cancer patients. *Semin. Oncol.*, **17,** 49–53.

CLARK A AND FALLOWFIELD LJ. (1986). Quality of life measurements in patients with malignant disease: a review. *J. R. Soc. Med.*, **79,** 165–169.

COMIS RL. (1993). Extensive small cell lung cancer. *Lung Cancer*, **9(suppl. 1),** S27–S39.

EARL HM, RUDD RM, SPIRO SG, ASH CM, JAMES LE, LAW CS, TOBIAS JS, HARPER PG, GEDDES DM, ERAUT D, PARTRIDGE MR AND SOUHAMI RL. (1991). A randomised trial of planned versus as required chemotherapy in small cell lung cancer: A Cancer Research Campaign trial. *Br. J. Cancer*, **64,** 566–572.

FAYERS PM, BLEEHEN NM, GIRLING DJ AND STEPHENS RJ. (1991). Assessment of quality of life in small-cell lung cancer using a daily diary card developed by the Medical Research Council Lung Cancer Working Party. *Br. J. Cancer*, **64,** 299–306.

FELD R, EVANS WK, COY P, HODSON I, MACDONALD AS, OSOBA D, PAYNE D, SHELLEY W AND PATER JL. (1987). Canadian multicenter randomized trial comparing sequential and alternating administration of two non-cross-resistant chemotherapy combinations in patients with limited small-cell carcinoma of the lung. *J. Clin. Oncol*, **5,** 1401–1409.

FIGUEREDO AT, HRYNIUK WM, STRAUTMANIS I, FRANK G AND RENDELL S. (1985). Co-trimoxazole prophylaxis during high-dose chemotherapy of small cell lung cancer. *J. Clin. Oncol.*, **3,** 54–64.

GEDDES DM, DONES L, HILL E, LAW K, HARPER PG, SPIRO SG, TOBIAS JS AND SOUHAMI RL. (1990). Quality of life during chemotherapy for small cell lung cancer: Assessment and use of a daily diary card in a randomized trial. *Eur. J. Cancer*, **26,** 484–492.

HAYBITTLE JL. (1979). The reporting of non-significant results in clinical trials. In *Clinical Trials in Early Breast Cancer*, Scherulen H R, Weckesser G, Armbruster I. (eds), Springer: Berlin.

HRYNIUK WM. (1988). The importance of dose intensity in the outcome of chemotherapy. In *Important Adv. Oncol.*, De Vita VT Jnr, Hellman S and Rosenberg SA. (eds.) pp. 121–141. Lippincott: Philadelphia.

IHDE DC, MULSHINE JL, KRAMER BS, STEINBERG SM, LINNOILA RI, GAZDAR AF, EDISON M, PHELPS RM, LESAR M, PHARES JC, GRAYSON J, MINNA JD AND JOHNSON BE. (1994). Prospective randomized comparison of high-dose and standard-dose etoposide and cisplatin chemotherapy in patients with extensive-stage small-cell lung cancer. *J. Clin. Oncol.*, **12,** 2022–2034.

KLASA RJ, MURRAY N AND COLDMAN AJ. (1991). Dose-intensity meta-analysis of chemotherapy regimens in small-cell carcinoma of the lung. *J. Clin. Oncol.*, **9,** 499–508.

MEDICAL RESEARCH COUNCIL LUNG CANCER WORKING PARTY. (1993). A randomised trial of three or six courses of etoposide, cyclophosphamide, methotrexate and vincristine or six courses of etoposide and ifosfamide in small cell lung cancer (SCLC) I: survival and prognostic factors. *Br. J. Cancer*, **68,** 1150–1156.

MILES DW, EARL HM, SOUHAMI RL, HARPER PG, RUDD R, ASH CM, JAMES L, TOBIAS JS AND SPIRO SG. (1991). Intensive weekly chemotherapy for good-prognosis patients with small-cell lung cancer. *J. Clin. Oncol.*, **9,** 280–285.

PETO R, PIKE MC, ARMITAGE P, BRESLOW NE, COX DR, HOWARD SV, MANTEL N, MCPHERSON K, PETO J AND SMITH PG. (1977). Design and analysis of randomized clinical trials requiring prolonged observation of each patient. *Br. J. Cancer*, **35,** 1–39.

RAWSON NSB AND PETO J. (1990). An overview of prognostic factors in small cell lung cancer. A report from the Subcommittee for the Management of Lung Cancer of the UKCCCR. *Br. J. Cancer*, **61,** 597–604.

SCULIER JP, PAESMANS M, BUREAU G, DABOUIS G, LIBERT P, VANDERMOTEN G, VAN CUTSEM O, BERCHIER MC, RIES F AND MICHEL J. (1993). Multiple-drug weekly chemotherapy versus standard combination regimen in small-cell lung cancer: A phase III randomized study conducted by the European Lung Cancer Working Party. *J. Clin. Oncol.*, **11,** 1858–1865.

SLEVIN ML, PLANT H, LYNCH D, DRINKWATER J AND GREGORY WM. (1988). Who should measure quality of life, the doctor or the patient? *Br. J. Cancer*, **57,** 109–12.

SOUHAMI RL AND LAW K. (1990). Longevity in small cell lung cancer. *Br. J. Cancer*, **61,** 584–589.

SOUHAMI RL, BRADBURY I, GEDDES DM, SPIRO SG, HARPER PG AND TOBIAS JS. (1985). Prognostic significance of laboratory parameters measured at diagnosis in small cell carcinoma of the lung. *Cancer Res.*, **45,** 2878–2882.

SOUHAMI RL, RUDD R, RUIZ DE ELVIRA M-C, JAMES L, GOWER N, HARPER PG, TOBIAS JS, PARTRIDGE MR, DAVISON AG, TRASK C AND SPIRO SG. (1994). Randomised trial comparing weekly versus 3-week chemotherapy in small-cell lung cancer: A Cancer Research Campaign Trial. *J. Clin. Oncol.*, **12,** 1806–1813.

TANNOCK IF, BOYD NF, DE BOER G, ERLICHMAN C, FINE S, LAROCQUE G, MAYERS C, PERRAULT D AND SUTHERLAND H. (1987). A randomized trial of two dose levels of cyclophosphamide, methotrexate, and fluorouracil chemotherapy for patients with metastatic breast cancer. *J. Clin. Oncol.*, **6,** 1377–1387.